# Genome-wide analysis and characterization of *Aux/IAA* family genes related to fruit ripening in papaya (*Carica papaya* L.)

**DOI:** 10.1186/s12864-017-3722-6

**Published:** 2017-05-05

**Authors:** Kaidong Liu, Changchun Yuan, Shaoxian Feng, Shuting Zhong, Haili Li, Jundi Zhong, Chenjia Shen, Jinxiang Liu

**Affiliations:** 1 Life Science and Technology School, Lingnan Normal University, Zhanjiang, Guangdong 524048 China; 2 College of Life and Environmental Science, Hangzhou Normal University, Hangzhou, 310036 China

**Keywords:** Auxin, Aux/IAA family, Papaya, Expression profile, Fruit ripening

## Abstract

**Background:**

*Auxin/indole-3-acetic acid* (Aux/IAA) family genes encode short-lived nuclear proteins that mediate the responses of auxin-related genes and are involved in several plant developmental and growth processes. However, how *Aux/IAA* genes function in the fruit development and ripening of papaya (*Carica papaya* L.) is largely unknown.

**Results:**

In this study, a comprehensive identification and a distinctive expression analysis of 18 *C. papaya Aux/IAA* (*CpIAA*) genes were performed using newly updated papaya reference genome data. The *Aux/IAA* gene family in papaya is slightly smaller than that in *Arabidopsis*, but all of the phylogenetic subfamilies are represented. Most of the *CpIAA* genes are responsive to various phytohormones and expressed in a tissues-specific manner. To understand the putative biological functions of the *CpIAA* genes involved in fruit development and ripening, quantitative real-time PCR was used to test the expression profiling of *CpIAA* genes at different stages. Furthermore, an IAA treatment significantly delayed the ripening process in papaya fruit at the early stages. The expression changes of *CpIAA* genes in ACC and 1-MCP treatments suggested a crosstalk between auxin and ethylene during the fruit ripening process of papaya.

**Conclusions:**

Our study provided comprehensive information on the Aux/IAA family in papaya, including gene structures, phylogenetic relationships and expression profiles. The involvement of *CpIAA* gene expression changes in fruit development and ripening gives us an opportunity to understand the roles of auxin signaling in the maturation of papaya reproductive organs.

**Electronic supplementary material:**

The online version of this article (doi:10.1186/s12864-017-3722-6) contains supplementary material, which is available to authorized users.

## Background

Auxin, the most ubiquitous phytohormone in plants, is involved in various biological processes, such as organ development, fruit ripening, responses to environmental stimuli and phototropism [1, 2]. At an early stage in auxin signal transduction, several gene families, including Aux/IAA (auxin/indole-3-acetic acid), GH3 (Gretchen Hagen3) and SAUR (small auxin up RNA), respond to auxin treatments [3]. The Aux/IAA genes represent a classical auxin-responsive gene family that are mostly rapidly induced by auxin [4]. The first Aux/IAA family member was isolated in soybean and then was identified in many different plant species [5–7]. As an important component of the auxin signaling pathway, Aux/IAA proteins are involved in the expression regulation of a large number genes downstream of auxin signaling through the release of auxin response factors (ARFs) [8].

Most of the Aux/IAA proteins contain four conserved domains, I, II, III and IV [9, 10]. Briefly, domain I is a repressor domain that contains a leucine repeat motif (LxLxL) [11]. Recent studies found that the TOPLESS (TPL) protein mediates auxin-dependent transcriptional repression by interacting with domain I of an Aux/IAA protein [12]. Domain II is a key component responsible for the instability of Aux/IAA proteins and is recognized by the ubiquitin-proteasome protein (TIR1) degradation pathway. Domains III and IV are the binding sites for the formation of Aux/IAA-ARF hetero-dimerization [13, 14].

Due to the functional redundancy among family members, phenotypes associated with a loss of function in Aux/IAA genes are scarce. Based on the characterizations of gain-of-function mutants in model plants, the diverse roles of Aux/IAAs in plants have been well elucidated. In *Arabidopsis*, the *iaa16-1* mutation, a dominant gain-of-function mutation in IAA16, reduces the sensitivity to both auxin and abscisic acid (ABA) [15]. Domain II mutations in other AUX/IAAs affect various aspects of plant growth and development, including lateral root formation, gravitropism, phototropism, pollination and adventitious formation [16].

Recently, many studies have revealed the involvement of Aux/IAA proteins in the development of reproductive organs. In *Arabidopsis*, the over-expression of IAA1 with a domain II mutation damages cell elongation and cell division in inflorescences [17]. In maize, two Aux/IAA proteins, BIF1 and BIF4, control inflorescence architecture by regulating the expression of BARREN STALK1, which is a basic helix-loop-helix transcriptional regulator necessary for axillary meristem formation, that shows a striking boundary expression pattern [18]. Due to the minimal level of auxin signaling, the inflorescences of a dominant IAA7 mutant, *axr2*, display negative phototropism, with a similar response curve to the positive phototropism of *Arabidopsis* wild-type stems [19]. In tomato, SlIAA9 is a negative auxin response regulator and its down-regulation triggers fruit set before pollination [20]. Sl-IAA27, another Aux/IAA protein that is closely related to SlIAA9 in terms of sequence homology, regulates fruit initiation and development in a distinct manner [21].

As important components of the auxin signaling pathway, Aux/IAA family proteins have been identified in many plant species. Thus far, 34 Aux/IAA family members in *Arabidopsis*, 26 members in tomato (*Solanum lycopersicon*), 34 members in maize (*Zea mays* L.), 17 members in *Medicago* (*Medicago truncatula*), 27 members in cucumber (*Cucumis sativus* L.), and 31 members in rice (*Oryza sativa* L.) have been identified [10, 22–26]. As a climacteric fruit, the market value of papaya is significantly limited by its short-term shelf life and rapid softening [27]. Fruit ripening is associated with auxin signaling involved in the postharvest storage of fruits [28]. The elucidation of how AUX/IAA-mediated auxin signaling function in postharvest decay is therefore of importance to both plant biologists and agronomists [29]. In the present work, we provide comprehensive information on the genomic structures, sequence homology levels and expression patterns of *Aux/IAA* genes in papaya. Our studies provide a new insight into the complexity of papaya Aux/IAA expression during the fruit ripening process. The distinct spatio-temporal expression patterns of papaya *Aux/IAA* (henceforth referred to as *CpIAA*) genes, and their differential responses to ripening-related hormones, provide clues for the functional characterizations of the auxin-responsive genes involved in fruit development and ripening.

## Methods

### Plant material, growth conditions and hormone

Two-year-old *C. papaya* cv. ‘Sunrise’ trees were planted in a 3 m × 3 m plot with drip irrigation at the field experimental station (Match 2014) in Lingnan Normal University, Zhanjiang, China. The distance among trees are: 2 m between rows and 1.5 m along rows. Papaya fruits at the color break stage (5% ≤ peel color ≤ 15% yellow) were harvested in October 2014. The selected fruit were washed with deionized water, and then dipped in 75% (w/w) alcohol for 45 s to eliminate potential microbes. This station has a gentle tropical oceanic monsoon climate with an average daily temperature of 22.8°C, minimum temperature of 15.7°C and maximum temperature of 28.8°C. The total yearly rainfall ranges between 1,100 and 1,800 mm [30]. The environmental conditions were strictly recorded during the sampling period.

Five tissue samples were used for tissue-specific expression pattern analysis. In detail, the shoot, leaf, root samples were selected from 2-years-old papaya trees. The fruit samples were harvested from fruits at the color break stage (5% ≤ peel color ≤ 15% yellow) of two-old-year trees. The flower samples were selected from mature flower with opened petals of two-old-year trees.

Fruit samples were soaked in liquid Murashige and Skoog (MS) medium with various hormone treatments, including 10 μM IAA for 3 h, 10 μM salicylic acid (SA) for 3 h, 10 μM abscisic acid (ABA) for 3 h and 10 μM gibberellic acid for 3 h. For ethylene regulation treatments, 1-aminocyclopropane-1-carboxylic acid (ACC) and 1-methylcyclopropene (1-MCP) treatments were included. For ACC treatment, papaya fruits were dipped into 10 μM ACC solutions for 3 h and then taken out to be air-dried at room temperature. For 1-MCP treatment, papaya fruits were incubated with 300 nL L^−1^ of 1-MCP, which was calculated from the active ingredient, for 16 h in the airtight containers. Untreated fruits were used as controls. Samples from each treatment were collected, and the total RNA was isolated for qRT-PCR analysis.

The papaya fruit samples at different developmental stages were harvested at 20, 40, 60, 80, 100 and 120 days after anthesis. The time points after harvest of 0, 5, 10, 15, 20 and 25 d were defined as postharvest stage 1–6, respectively. For each fruit sample, the fruit kernel was excluded, and the sarcocarp with the pericarp was chopped up, frozen in liquid nitrogen and stored at −80°C for further experiments. To avoid environmental effects, the fruits were collected from different places in the field.

### RNA isolation and quantitative real time PCR (qRT-PCR)

Total RNA from different organs, such as shoots, leaves, flowers, fruits and roots, was extracted using a Plant RNeasy Mini kit (Qiagen, Hilden, Germany) according to the manufacturer’s instructions [31]. To test the expression of *CpIAA* genes during different fruit developmental, 30 fruits were divided into six groups (five fruits in each group). To test the expression of CpIAA genes during different fruit ripening stages, another 30 fruits were divided into six groups (five fruits in each group).

Genomic DNA contamination in total RNA was removed by DNase I. The qRT-PCR analysis was performed as previously described [32]. To visualize the qRT-PCR data, a heat map was constructed by MeV software using the average *Ct* values. The *CpActin* gene was used as an internal standard to calculate relative fold-differences based on comparative cycle threshold (2^-ΔΔ*Ct*^) values [33]. All of the primer sequences are listed in Additional file 1.

### Genome-wide identification of *CpIAA* genes

*Arabidopsis* IAA protein sequences were used to search against the *C. papaya* proteome database on Phytozome 11.0.2 using the TBLASTN algorithm (http://phytozome.jgi.doe.gov). The hidden Markov model profile of the Aux/IAA protein family (Pfam: 02309 AUX/IAA family) was employed to identify the *Aux/IAA* genes from the papaya genome. All of the obtained sequences were sorted as unique genes for a detailed analysis.

### Phylogenetic tree building, gene structure and motif prediction

A multiple sequence alignment was performed with the full length sequences of *CpIAA* proteins using ClustalW and the default parameters. A phylogenetic tree was constructed with the aligned AtIAA protein sequences and CpIAA protein sequences using MEGA5.1 (http://www.megasoftware.net/) employing the neighbor-joining method. Bootstrap values were calculated from 1,000 iterations. The predictions of four classical domains (I, II, III and IV) in CpIAA proteins was performed by the software MEGA 5.1. The DNA and cDNA sequences corresponding to each predicted gene were obtained from Phytozome 11.0.2, and the intron distribution patterns of the *CpIAA* genes were analyzed by the software GSDS (http://gsds.cbi.pku.edu.cn/) [34].

### Analysis of hormone-related *cis*-elements

The promoter regions (1,500 bp before ATG) of the *CpIAA* genes were scanned for several hormone-related *cis*-elements, including the ABA-responsive element (ABRE), gibberellin-responsive element (GARE), SA-responsive element (SARE), and auxin-responsive element (AuxRE). Furthermore, the results were confirmed by the software PLACE (http://www.dna.affrc.go.jp/).

### Statistical analysis

Differences between values were calculated using Student’s *t*-test at a significance level of 0.05 in Microsoft Excel software. All of the expression analyses were performed for five biological replicates, and the values shown in the figures represent the average values of five replicates.

## Results

### Genome-wide identification of *CpIAA* genes

The new version (v0.4) of the papaya genome, which is approximately 135 Mb arranged in 4,114 contigs with at least one gene model, was used to identify 18 *IAA* genes in *C. papaya*. The scores of the search results for CpIAA proteins are listed in Additional file 2. All of these genes were named according to the phylogenetic relationship between *C. papaya* and the model plant *Arabidopsis*. Detailed information on these *CpIAA* genes, including gene names, locus IDs, open reading frame (ORF) lengths, intron numbers and deduced polypeptide basic parameters, is listed in Additional file 3.

### Protein structure, gene structure and phylogenetic relationship analysis of *IAA* family genes

**Fig. 1:**
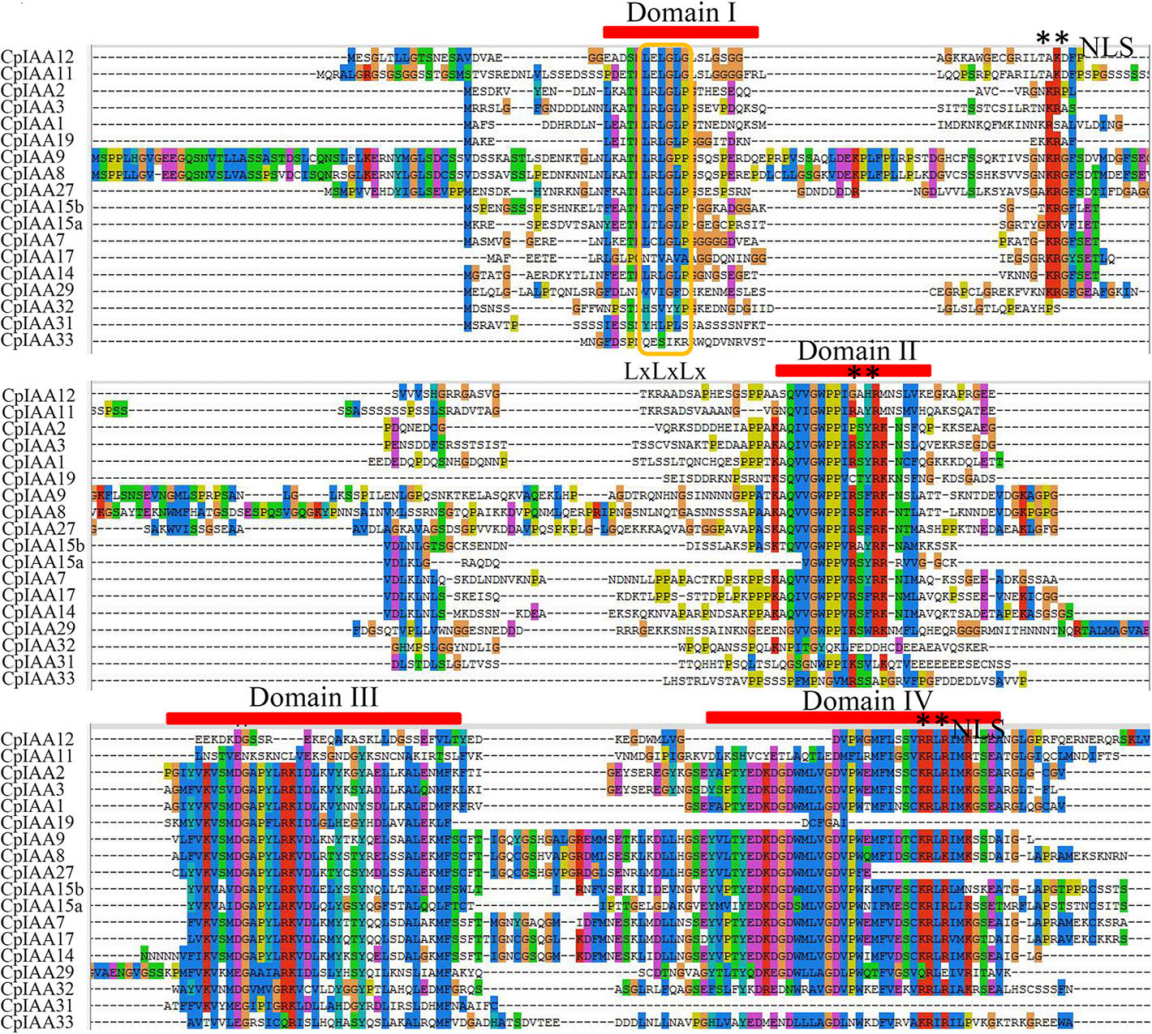
Protein domain analysis of CpIAA family members. The alignment of papaya IAA proteins obtained with the ClustalW program. The multiple alignment of the domains I–IV of the papaya IAA proteins are indicated by *red* lines. Colored shading indicates identical and conserved amino acid residues. The LxLxLx motif is denoted by a thin *yellow* box. The two NLSs are marked by *black* asterisks

The multiple sequence alignment results showed that four conserved domains (I, II, III and IV) were contained in most CpIAA proteins. A classical LxLxLx motif was identified in domain I of most of the CpIAA proteins, except CpIAA17, CpIAA29 and CpIAA31-33. Two nuclear localization signals (NLSs), a bipartite NLS and a Simian virus 40-like NLS, were found in most of the identified CpIAA proteins. The bipartite NLS, containing two stretches of lysine/arginine residues, was found between domains I and II, while the Simian virus 40-like NLS, consisting of several positively charged amino acid residues, was found in domain IV [22]. These putative NLSs indicate that CpIAAs are nuclear-located proteins (Fig. 1). The distribution pattern of the exons and introns for each *CpIAA* gene was analyzed by a comparison of the full-length transcript sequences with the corresponding genomic DNA sequences. The number of introns varied from one to four in the CpIAA family and is similar to the numbers found in other plant species [24, 25, 35] (Additional file 4).

**Fig. 2:**
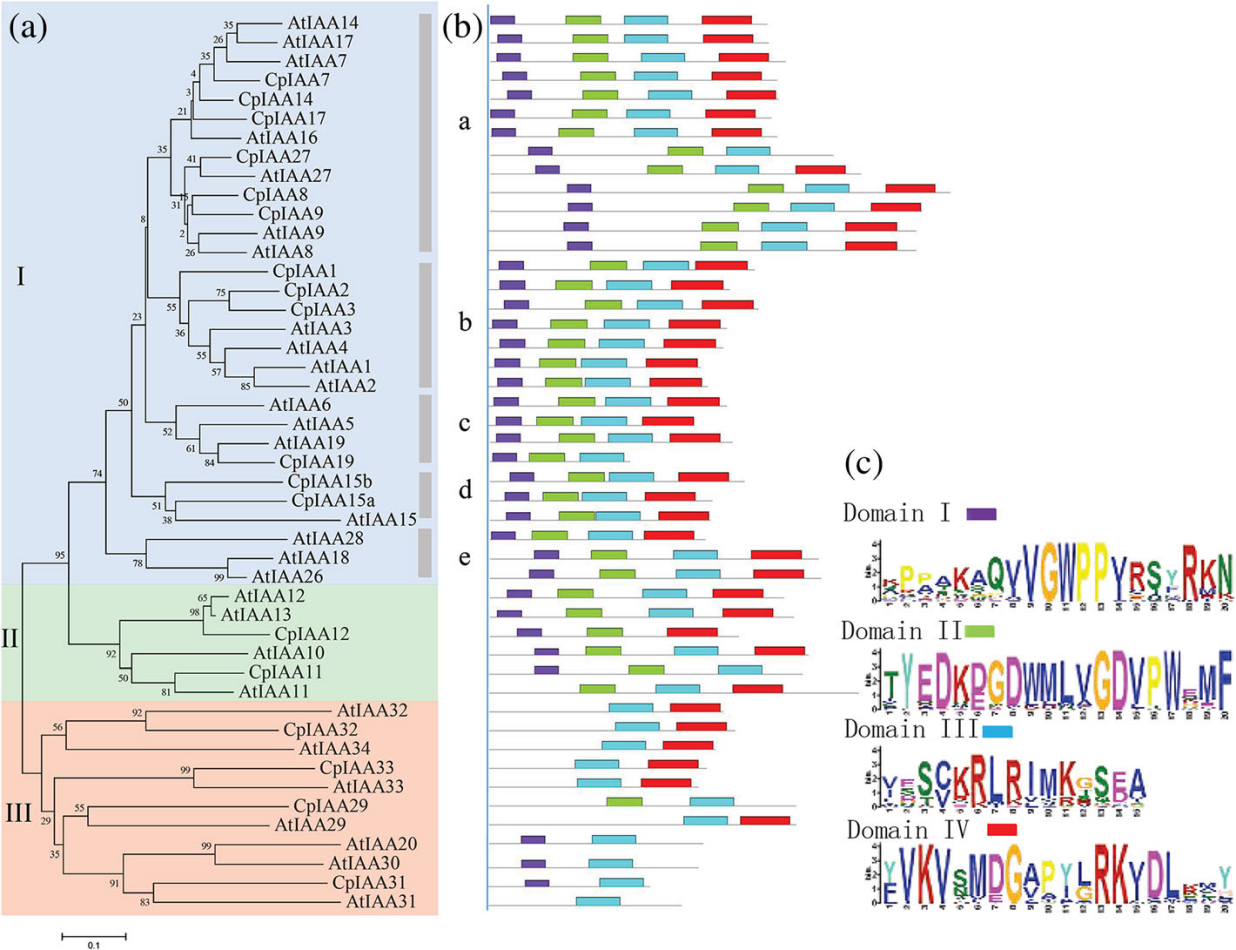
Phylogenetic relationships and motif distribution analysis. **a** Phylogenetic relationships between *Arabidopsis* and papaya IAA families. The unrooted tree was generated using the MEGA5.1 program by the neighbor-joining method. Bootstrap support from 1,000 replicates are shown at each branch. **b** The motif distribution in *Arabidopsis* and papaya IAA proteins. Motifs of Aux/IAA proteins were analyzed using the MEME web server. Four motifs, representing the domains I, II, III and IV, are mapped on all of the Aux/IAA proteins in different colors. **c** The height of each box represents the specific amino acid conservation in each motif

To explore the phylogenetic relationships of the IAA proteins between papaya and the model plant *Arabidopsis*, a phylogenetic tree was constructed, including 31 members in *Arabidopsis* and 18 members in *C. papaya* [36]. An unrooted phylogenetic tree was generated by the alignments of these IAA protein sequences. The phylogenetic tree grouped all of the IAAs into three major classes, Groups I, II and III, with well-supported bootstrap values. Group I was further divided into five subgroups, a, b, c, d and e (Fig. 2a).

The Multiple Expectation Maximization for Motif Elicitation (MEME) web server (http://meme-suite.org/) was used to analyze the domain distributions in *C. papaya* and *Arabidopsis* IAA proteins. Four different conserved domains were mapped to all of the IAA proteins, as seen in Fig. 2b. Interestingly, most of the IAA proteins containing the four domains belonged to Groups I and II, while most of the IAA proteins with truncated domains were classed into Group III. For the CpIAA proteins, ‘domain I’ is missing in CpIAA29, CpIAA32 and CpIAA33; ‘domain II’ is missing in CpIAA31, CpIAA32 and CpIAA33; only CpIAA12 does not contain ‘domain III’; and ‘domain IV’ is missing in CpIAA11, CpIAA19, CpIAA27 and CpIAA31 (Fig. 2b).

### Analysis of hormone-related *cis*-elements in the promoter regions of *CpIAA* and *AtIAA* genes

**Fig. 3:**
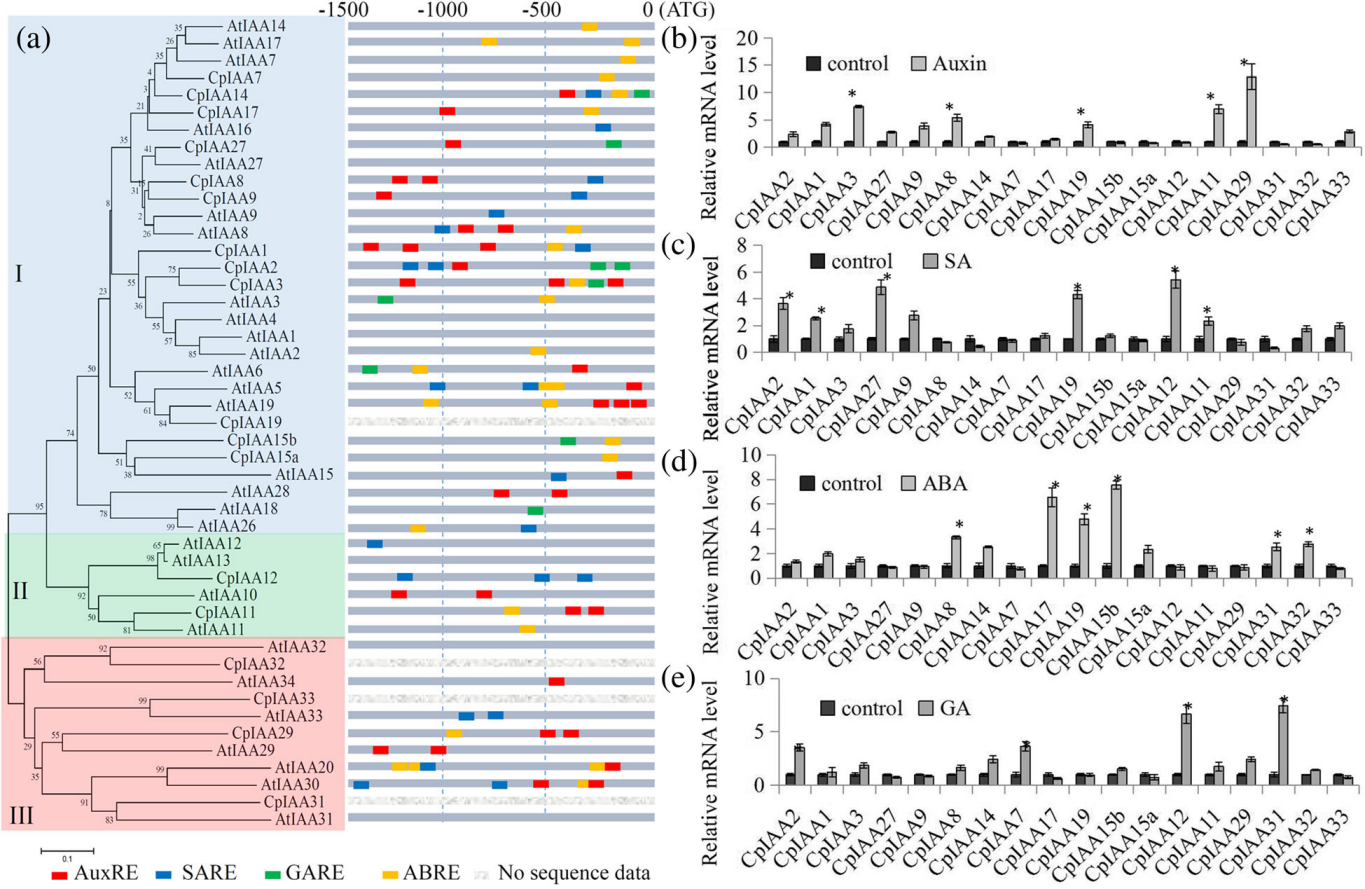
Analysis of specific *cis*-elements in promoters, and the expression patterns of *CpIAAs* and *AtIAAs* under various hormone treatments. **a** The 1,500-bp promoter sequences of corresponding *CpIAA* and *AtIAA* genes were used to analyze specific hormone-related cis-elements, including AuxRE, SARE, GARE and ABRE, which are color coded. The expression of the *CpIAA* genes in response to (**b**) Auxin, (**c**) SA, (**d**) GA and (**e**) ABA treatments was analyzed by qRT-PCR. The expression levels of *CpIAA* genes in control seedlings were set to a value of 1. The expression levels of *CpIAA* genes in IAA- (100 μM), SA- (100 μM), GA- (100 μM) and ABA- (100 μM) treated seedlings were compared to a mock treatment as relative mRNA levels. Error bars represent standard deviations from five biological replicates. Significant differences (*P* < 0.05) between the control and hormone-treated samples are indicated by an asterisk

Some *cis*-elements that are involved in hormone-related gene expression regulation have been identified in plants, including ABRE, AuxRE, SARE and GARE [3]. We scanned the 1,500-bp upstream promoter regions found in most of the *CpIAA* and *AtIAA* genes with five important hormone-related *cis*-elements to gain insights into how the expression levels of *CpIAA* and *AtIAA* genes were responsive to hormone stimuli. Several hormone-related *cis*-elements were enriched in the promoter regions of *CpIAA* genes. Interestingly, the promoters of *CpIAA1*, *CpIAA2* and *CpIAA3* contained many hormone-related *cis*-elements (more than five elements). One AuxRE, two SAREs and two GAREs were contained in the promoter of *CpIAA2*; three AuxREs, one ABRE and one SARE were contained in the promoter of *CpIAA1*; and three AuxREs, one ABRE and one GARE were contained in the promoter of *CpIAA3* (Fig. 3a). For AtIAA family genes, the four hormone-related *cis*-elements were enriched in the promoter regions. The promoters of *AtIAA19*, *AtIAA20* and *AtIAA30* contained many hormone-related *cis*-elements (more than five elements). Three AuxREs and two ABREs were contained in the promoter of *AtIAA19*; One AuxREs, three ABRE and one SARE were contained in the promoter of *AtIAA20*; and two AuxREs, one ABRE and two SARE were contained in the promoter of *CpIAA3* (Fig. 3a). The numbers of stress-related *cis*-elements in the upstream 1.5-kb regions of the *CpIAA* and *AtIAA* family genes are listed in Additional file 5.

### Expression of *CpIAA* and *AtIAA* genes in response to auxin, ABA, SA and GA treatments

Auxin, ABA, SA and GA are four major hormones involved in fruit development and ripening [37–39]. The expression levels of *CpIAA* genes were tested in the *C. papaya* fruits by qRT-PCR under auxin, ABA, SA and GA treatments. The expression levels of *CpIAA3*, *CpIAA8, CpIAA11* and *CpIAA29* were significantly up-regulated over five-fold under the auxin treatment, while *CpIAA31* and *CpIAA32* were significantly reduced by the auxin treatment (Fig. 3b). Under the SA treatment, *CpIAA2*, *CpIAA27*, *CpIAA19* and *CpIAA12* were significantly induced, while *CpIAA14* and *CpIAA31* were significantly down-regulated (Fig. 3c). Under the GA treatment, *CpIAA2*, *CpIAA7*, *CpIAA12* and *CpIAA31*, were significantly induced and no *CpIAA* gene was significantly reduced (Fig. 3d). The expression levels of *CpIAA17*, *CpIAA19* and *CpIAA15b* were up-regulated under ABA treatment (Fig. 3e).

The expression levels of *AtIAA* genes response to various hormones was publicly available. Furthermore, the expression of *AtIAA* genes in response to Auxin, SA, ABA and GA were searched at NCBI in the dataset GSE39384. Interestingly, more than two AuxREs were contained in the promoter regions of *AtIAA19*, *AtIAA29* and *AtIAA30*, and the expression of *AtIAA19*, *AtIAA29* and *AtIAA30* were significantly up-regulated by auxin treatment. Few GAREs were contained in the promoter regions of AtIAA family gene, and the expression of most *AtIAA* family genes showed no changes to GA treatment (Additional file 6).

### *CpIAA* gene expression patterns in different *C. papaya* tissues

**Fig. 4:**
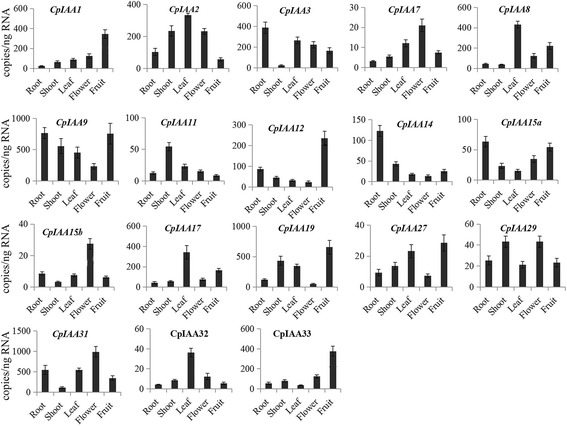
Tissues-specific expression patterns of *CpIAA* genes. The expression patterns of the *CpIAA* genes in the five indicated organs were analyzed by absolute qRT-PCR. The data were analyzed by five independent replicates, and the standard deviations are shown with error bars

There is a close relationship between biological functions and the organ-specific expression patterns of *CpIAA* genes. In this study, the spatio-specific expression of each member of the CpIAA family was assessed in various tissues, such as roots, flowers, shoots, leaves and fruits. The *CpIAA* gene transcript accumulations were detectable in four different organs. However, some *CpIAA* genes displayed a clear preferential expression in a specific organ, such as *CpIAA11* showing the highest expression level in shoot, *CpIAA8*, *CpIAA17* and *CpIAA32* in leaf, *CpIAA14* in root, *CpIAA7* and *CpIAA15b* in flower, and *CpIAA1*, *CpIAA12* and *CpIAA33* in fruit (Fig. 4). Overall, the organ-preferential expression displayed by some *CpIAA* genes could be indicative of their involvement in specific *C. papaya* tissues and developmental processes.

### Expression of *CpIAA* genes at different fruit developmental and ripening stages

**Fig. 5:**
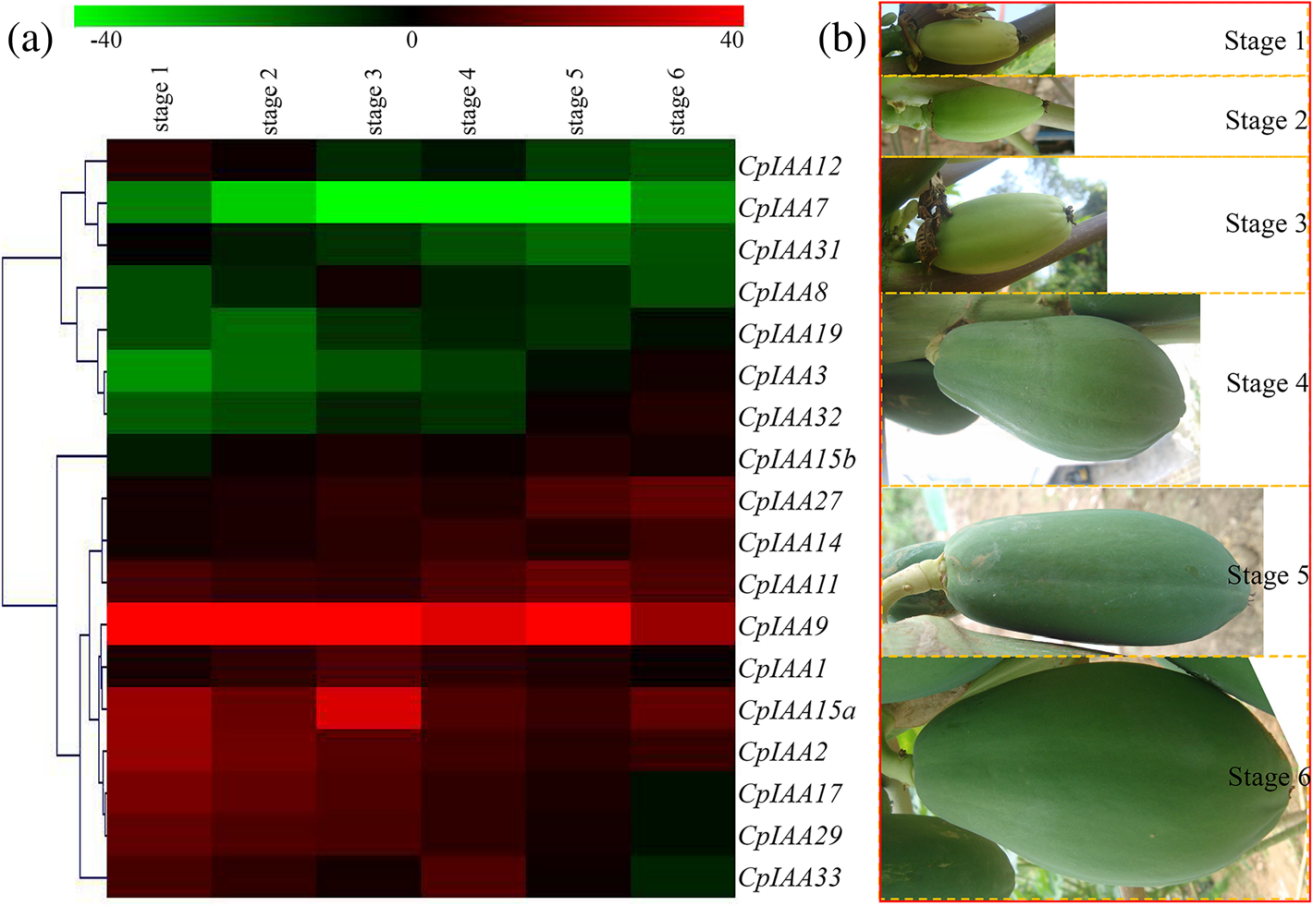
Heatmap of *CpIAA* gene expression during different fruit developmental stages. **a** Changes in the expression levels during different fruit developmental stages, which are schematically depicted above the displayed qRT-PCR data, are relative to RNA accumulation levels. Levels of down-regulated expression (*green*) or up-regulated expression (*red*) are shown on a log2 scale from the highest to the lowest expression for each *CpIAA* gene. Significant (*P* < 0.05) differences are indicated by an asterisk. **b** Different fruit developmental stages are shown as pictures

Several genetic studies have uncovered that fruit development and ripening is an auxin-related process [40]. Firstly, we analyzed the expression levels of *CpIAA* genes during six different developmental stages. Several *CpIAA* genes, including *CpIAA19*, *CpIAA3*, *CpIAA32* and *CpIAA15b*, were induced during the developmental process and some *CpIAA* genes, including *CpIAA17*, *CpIAA29* and *CpIAA33*, were reduced (Fig. 5a). The six different developmental stages are shown in Fig. 5b.

**Fig. 6:**
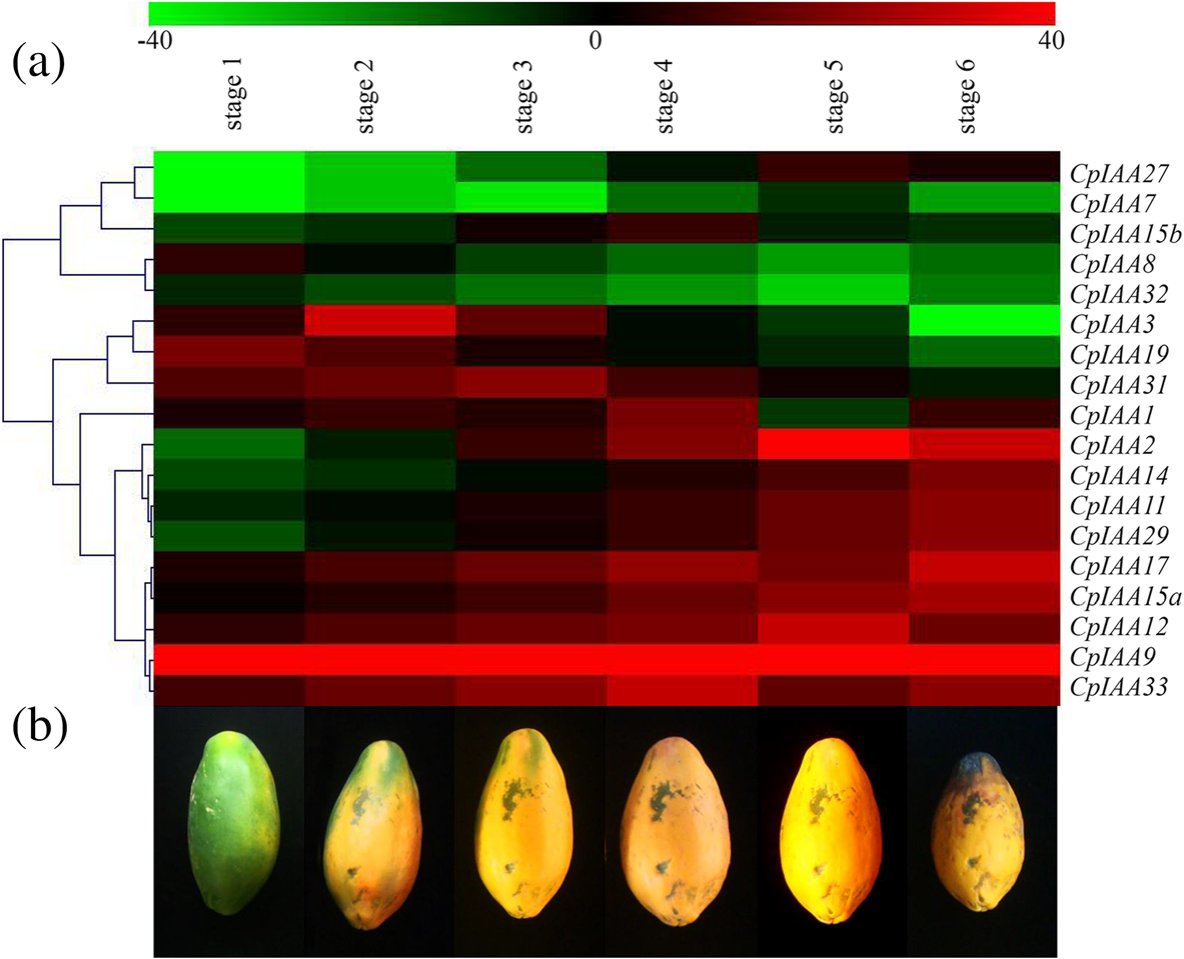
Heatmap of *CpIAA* gene expression during different fruit ripening stages. **a** Changes in the expression levels during different fruit ripening stages, which are schematically depicted above the displayed qRT-PCR data, are relative to RNA accumulation levels. Levels of down-regulated expression (*green*) or up-regulated expression (*red*) are shown on a log2 scale from the highest to the lowest expression for each *CpIAA* gene. Significant (*P* < 0.05) differences are indicated by an asterisk. **b** Different fruit ripening stages are shown as pictures

To elucidate the functions of the *CpIAA* genes during the fruit ripening period, their expression levels at six different postharvest stages were analyzed. The expression levels of most of the *CpIAA* genes were significantly changed during fruit ripening and softening. *CpIAA3*, *CpIAA19* and *CpIAA31* expression levels significantly increased, while *CpIAA2, CpIAA11, CpIAA14* and *CpIAA29* expression levels significantly decreased. The expression of *CpIAA*27 peaked at Stage 5, and then declined slightly at Stage 6. The expression of *CpIAA15b* was induced significantly at Stage 3, and reached its peak at Stage 4 (Fig. 6).

### Expression of *CpIAA* genes under ACC and 1-MCP treatments

**Fig. 7:**
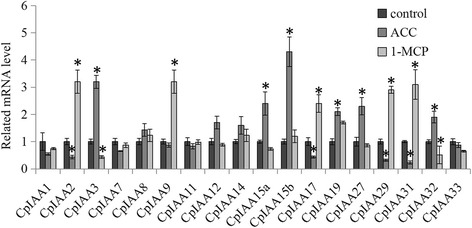
The expression patterns of *CpIAA* under ACC and 1-MCP treatments. The expression levels of *CpIAA* genes in ACC and 1-MCP treated seedlings were compared to a mock treatment as relative mRNA levels. Error bars represent standard deviations from five biological replicates. Significant differences (*P* < 0.05) between the control and hormone-treated samples are indicated by an asterisk

Ethylene is a major hormone that enhances fruit ripening. In our study, an ethylene precursor, ACC, and an ethylene inhibitor, 1-MCP, were used to test how the expression of *Aux/IAA* family genes was involved in fruit ripening [41]. The data showed that the expression of CpIAA3, CpIAA15a, CpIAA15b, CpIAA19, CpIAA27 and CpIAA32 was significantly induced by ACC treatment; while the expression of CpIAA2, CpIAA9, CpIAA17, CpIAA29 and CpIAA31 was significantly up-regulated by 1-MCP treatment. Four genes, including CpIAA2, CpIAA17, CpIAA29 and CpIAA31, were significantly reduced under ACC treatment; while two genes, CpIAA3 and CpIAA32, were significantly down-regulated under 1-MCP treatment (Fig. 7).

## Discussion

Auxin is key signaling molecule for most growth and developmental processes in plants [42–45]. Aux/IAAs repress the expression of down-stream genes by binding to ARFs, which are also involved in auxin gene expression responses [46]. However, in papaya, there is little available information on *IAA* genes. The characterization and expression pattern analysis of *CpIAA* genes allowed us to uncover the mechanisms behind auxin involvement in fruit development and the ripening of papaya. In total, 18 *IAA* genes were identified in papaya, which was less than in model plants, such as *Arabidopsis* (34 members) and rice (31 members) [47].

In plants, the expression levels of *IAA* family genes was regulated by different hormones [35]. Our data showed that the transcript levels of many *CpIAA* genes were regulated by various hormone treatments (Fig. 3b–e). Consistent with the changes in *CpIAA* expression, the promoter analysis revealed the presence of several well-identified hormone response elements in the promoter regions of the majority of *CpIAA* genes (Fig. 3a). This suggested that crosstalk among various hormones existed in papaya.

Many *AtIAA* and *OsIAA* genes have been identified in previous reports [16, 48]. Comparative studies on phylogenetic relationships may provide useful information on the respective biological functions in papaya. Floral initiation is a major step in the life cycle of plants. In *Arabidopsis*, a gain-of-function mutation in IAA7 reduces the light-dependent gravitropism and phototropism of inflorescences, and confers late flowering under short-day light [46, 49]. Interestingly, *CpIAA7*, a orthologous gene of *AtIAA7*, was most highly expressed in flowers (Fig. 4), suggesting a putative role for *CpIAA7* in the flowering process of papaya. *AtIAA19* is highly expressed in stamen filaments, and its gain-of-function mutant, *msg2*, is defective in stamen filament development [50]. However, the expression of *CpIAA19*, a orthologous gene of AtIAA19, is very weak in flowers (Fig. 4). In addition, *AtIAA14* may be involved in both lateral and adventitious root formation [51]. The orthologous gene of *AtIAA14* in papaya, *CpIAA14*, was preferentially expressed in roots, indicating its importance in papaya root development.

We also built a phylogenetic tree to show the relationships of *IAA* genes between papaya and rice (Additional file 7). In rice, *OsIAA11* and *OsIAA23* both play essential roles in root system development [52, 53]. Based on the phylogenetic tree, the homologous gene of *OsIAA11* in papaya is *CpIAA17* and the homologous gene of *OsIAA23* in papaya is *CpIAA15b,* suggesting that these two genes may play roles in papaya root development.

Fruit development and ripening are complex developmental programs characterized by intense metabolic and textural changes [54]. Previous studies have presumed a close relationship between auxin and fruit development and ripening in various plant species [55]. Endogenous IAA plays roles in the flower and fruit development in papaya. However, how *IAA* genes function in the fruit development and ripening of papaya is largely unknown. In tomato, a model plant in fruit development studies, many *IAA* genes were identified as being involved in fruit development and ripening. For example, SlIAA17 regulates quality parameters during tomato fruit development by interacting with several ARF proteins [54, 56]. The expression of *CpIAA17*, a homologous gene of *SlIAA17* in papaya, was significantly reduced during the fruit developmental process, suggesting a putative function in papaya fruit development and ripening (Fig. 5). *SlIAA9*, an Aux/IAA family member in tomato, takes part in fruit development. *SlIAA9* showed constitutive expression in different organs and a rapid induction by auxin [6]. In our study, the expression of *CpIAA9*, the papaya homolog of *SlIAA9*, was also detectable in all of the tested organs and was induced significantly by an auxin treatment (Fig. 3b). Silencing *SlIAA27*, a gene closely related to *SlIAA9* in terms of sequence homology, causes a dramatic loss of fertility in tomato [21]. Here, the homologous gene of *SlIAA27* has been identified in papaya (*CpIAA27*). The expression of *CpIAA27* was significantly increased during the fruit ripening process and reached its peak at Stage 5 (Fig. 6). Our data indicated that *CpIAA9*, *CpIAA17* and *CpIAA27* may be candidate genes for further studies on papaya fruit ripening. On the other hand, it has been widely known that ethylene plays an important role during the ripening process of climacteric fruit [41]. Some studies showed that papaya displayed a dependence on ethylene, with an increase in production during its ripening process [57]. The significant changes in the expression of some CpIAA family genes under ACC and 1-MCP treatments indicated a crosstalk between auxin and ethylene during the ripening process of papaya fruits.

## Conclusion

In this study, comprehensive information on the Aux/IAA family in papaya, including gene structures, phylogenetic relationships and expression profiles, was provided. The involvement of *CpIAA* gene expression changes in fruit development and ripening gives us an opportunity to understand the roles of auxin signaling in the maturation of papaya reproductive organs.

## Additional files

Additional file 1: The primer sequences of papaya Aux/IAA family genes. (DOCX 11 kb)

Additional file 2: The scores of the search results for CpIAA proteins. (DOCX 12 kb)

Additional file 3: Aux/IAA family genes in *Carica papaya*. (DOCX 15 kb)

Additional file 4: Exon-intron structure analysis of *CpIAA* genes. (DOCX 35 kb)

Additional file 5: The numbers of stress-related *cis*-elements in the upstream 1.5-kb regions of the *CpIAA* and *AtIAA* family genes. (DOCX 13 kb)

Additional file 6 The expression levels of *AtIAA* genes response to various hormones. (DOCX 294 kb)

Additional file 7: The relationships of *IAA* genes between papaya and rice. (DOCX 77 kb)

## Abbreviations

ABA: Abscisic acid
ABRE: ABA-responsive element
ARFs: Auxin response factors
Aux/IAA: Auxin/indole-3-acetic acid
AuxRE: Auxin-responsive element
CpIAA: C. papaya Aux/IAA
GARE: Gibberellin-responsive element
GCC: Ethylene-responsive element
GH3: Gretchen hagen3
MEME: Multiple expectation maximization for motif elicitation
MS: Murashige and skoog
NLSs: Nuclear localization signals
ORF: Open reading frame
qRT-PCR: Quantitative real time PCR
SA: Salicylic acid
SARE: SA-responsive element
SAUR: Small auxin up RNA
TPL: TOPLESS
