# Supplementary material for: Genome-wide analysis and characterization of Aux/IAA family genes related to fruit ripening in papaya (Carica papaya L.)
Source: BMC Genomics. 2017 May 5;18:351. doi: 10.1186/s12864-017-3722-6 (PMC5420106; doi:10.1186/s12864-017-3722-6)
Supplement: Supplementary file 1 — The primer sequences of papaya Aux/IAA family genes. (DOCX 11 kb) [file 12864_2017_3722_MOESM1_ESM.docx]

**Additional file 1**: The primer sequences of papaya Aux/IAA family genes

| CpIAA1 | TGGATGGTGCTCCTTACTTG | CCTCTAGCCTCTGAGCCTTT |
| --- | --- | --- |
| CpIAA2 | GGCCGCCTATTCCATCTTAC | CTCCAACCAGCATCCAGTCA |
| CpIAA3 | TTTGGGATTCGGGAACGAC | TTGAGCCTTGGCAGGAGGT |
| CpIAA7 | ATCCATCAAAGCCACCTTCT | CGTCGGATAATTGCTGGTAA |
| CpIAA8 | GCCACTGTTTCCACTGCTTC | CCAGAGTTGCGGGATGATAG |
| CpIAA9 | GCGGGACCTGGTGTTCTATT | ACATCACCCACAAGCATCCA |
| CpIAA11 | TAGGAAGAGGGAGCGGTTCT | AAGAGGATGAGGGCGAAGAA |
| CpIAA12 | GGCTCACAGGATGAACAGCT | AGGTCCTCATTATGCGAAGC |
| CpIAA14 | AGGTTGGCTCCACCAGAACA | ACGGCTGTGACGATTCCTTT |
| CpIAA15a | GTCACCAGCGCCAACTATGA | CTGAAGAGCCGTGGAGAAGC |
| CpIAA15b | ATACGTGAAGGTGGCGGTAG | CCAGTTGCCTCTTTGCTGTT |
| CpIAA17 | GGTCTGCCAGGGAATACAGT | AACCGACAATTTGTGCCTTA |
| CpIAA19 | GGTGGTGGCAGGATGAATAT | CAAACATGGCGATCAAAGAG |
| CpIAA27 | ACTCGGGTCCTGTTGTGAAG | TCGTTAGTCTTGGGAGGGTG |
| CpIAA29 | GGTGGTGGCAGGATGAATAT | CAAACATGGCGATCAAAGAG |
| CpIAA31 | CCGTGGTCCCTAATGTCTTC | CTGGCTCCCTTGAATGAGTT |
| CpIAA32 | ATCCAGGCAAGGAAGACAAT | TTTGCGACCAACCATAACTC |
| CpIAA33 | CTGGCTTCGACGATGAGGAT | CAAGGTGACCAGGAACAGCA |
