# Supplementary material for: Genome-wide analysis and characterization of Aux/IAA family genes related to fruit ripening in papaya (Carica papaya L.)
Source: BMC Genomics. 2017 May 5;18:351. doi: 10.1186/s12864-017-3722-6 (PMC5420106; doi:10.1186/s12864-017-3722-6)
Supplement: Supplementary file 2 — The scores of the search results for CpIAA proteins. (DOCX 12 kb) [file 12864_2017_3722_MOESM2_ESM.docx]

| **Additional file 2**: The scores of the search results for CpIAA proteins | | | | |
| --- | --- | --- | --- | --- |
| Gene | Locus ID | bit-score | e-value | aligned regions |
| CpIAA1 | evm.TU.supercontig_1346.4 | 287.3 | 4.6E-69 | 6-164 |
| CpIAA2 | evm.TU.supercontig_58.36 | 201.1 | 3.5E-65 | 5-164 |
| CpIAA3 | evm.TU.supercontig_52.94 | 178.7 | 3.6E-56 | 8-164 |
| CpIAA7 | evm.TU.supercontig_58.37 | 125.9 | 1.5E-35 | 44-164 |
| CpIAA8 | evm.TU.supercontig_129.23 | 114.8 | 1.7E-30 | 35-164 |
| CpIAA9 | evm.TU.supercontig_2282.1 | 119 | 3.5E-32 | 48-164 |
| CpIAA11 | evm.TU.supercontig_87.29 | 35.8 | 0.0054 | 81-164 |
| CpIAA12 | evm.TU.supercontig_59.6 | 75.9 | 2.9E-17 | 35-164 |
| CpIAA14 | evm.TU.supercontig_1476.1 | 137.9 | 4.1E-40 | 7-164 |
| CpIAA15a | evm.TU.supercontig_23.159 | 101.7 | 6.9E-27 | 8-164 |
| CpIAA15b | evm.TU.supercontig_10.173 | 133.3 | 1.1E-38 | 7-164 |
| CpIAA17 | evm.TU.supercontig_52.93 | 122.5 | 3E-34 | 7-164 |
| CpIAA19 | evm.TU.supercontig_946.4 | 57 | 3.4E-11 | 9-109 |
| CpIAA27 | evm.TU.supercontig_12.32 | 84.3 | 5.6E-20 | 47-147 |
| CpIAA29 | evm.TU.supercontig_217.3 | 70.1 | 4.2E-15 | 81-160 |
| CpIAA31 | evm.TU.supercontig_57.25 | 55.8 | 3.4E-11 | 128-161 |
| CpIAA32 | evm.TU.supercontig_65.69 | 56.6 | 1.2E-10 | 81-164 |
| CpIAA33 | evm.TU.supercontig_233.11 | 40.8 | 0.00007 | 81-159 |
|  |  |  |  |  |
