# Supplementary material for: Genome-wide analysis and characterization of Aux/IAA family genes related to fruit ripening in papaya (Carica papaya L.)
Source: BMC Genomics. 2017 May 5;18:351. doi: 10.1186/s12864-017-3722-6 (PMC5420106; doi:10.1186/s12864-017-3722-6)
Supplement: Supplementary file 3 — Aux/IAA family genes in Carica papaya. (DOCX 15 kb) [file 12864_2017_3722_MOESM3_ESM.docx]

**Additional file 3:** Aux/IAA family genes in *Carica papaya*.

| Gene | Locus ID | ORF | No. of | Deduced polypeptide | | |
| --- | --- | --- | --- | --- | --- | --- |
|  |  | (bp) | introns | Length (aa) | Mol wt (kDa) | pI |
| CpIAA1 | evm.TU.supercontig_1346.4 | 636 | 3 | 211 | 23971.11 | 5.77 |
| CpIAA2 | evm.TU.supercontig_58.36 | 576 | 3 | 191 | 21532.46 | 5.58 |
| CpIAA3 | evm.TU.supercontig_52.94 | 645 | 3 | 214 | 23730.75 | 8.51 |
| CpIAA7 | evm.TU.supercontig_58.37 | 711 | 4 | 236 | 25751.51 | 6.63 |
| CpIAA8 | evm.TU.supercontig_129.23 | 1140 | 4 | 379 | 41062.33 | 7.9 |
| CpIAA9 | evm.TU.supercontig_2282.1 | 1066 | 4 | 356 | 38537.35 | 6.62 |
| CpIAA11 | evm.TU.supercontig_87.29 | 771 | 2 | 256 | 27048.36 | 8.94 |
| CpIAA12 | evm.TU.supercontig_59.6 | 609 | 4 | 202 | 21522.04 | 5.85 |
| CpIAA14 | evm.TU.supercontig_1476.1 | 714 | 3 | 237 | 25942.36 | 7.87 |
| CpIAA15a | evm.TU.supercontig_23.159 | 546 | 4 | 181 | 20131.99 | 7.65 |
| CpIAA15b | evm.TU.supercontig_10.173 | 624 | 4 | 207 | 22703.67 | 7.00 |
| CpIAA17 | evm.TU.supercontig_52.93 | 696 | 4 | 231 | 25429.24 | 8.37 |
| CpIAA19 | evm.TU.supercontig_946.4 | 343 | 0 | 115 | 12636.43 | 8.77 |
| CpIAA27 | evm.TU.supercontig_12.32 | 849 | 3 | 282 | 30010.77 | 5.27 |
| CpIAA29 | evm.TU.supercontig_217.3 | 756 | 3 | 251 | 27998.69 | 8.23 |
| CpIAA31 | evm.TU.supercontig_57.25 | 399 | 1 | 132 | 14443.04 | 5.03 |
| CpIAA32 | evm.TU.supercontig_65.69 | 606 | 3 | 201 | 22603.21 | 5.37 |
| CpIAA33 | evm.TU.supercontig_233.11 | 537 | 1 | 178 | 19782.38 | 6.19 |
