# Supplementary material for: Genome-wide analysis and characterization of Aux/IAA family genes related to fruit ripening in papaya (Carica papaya L.)
Source: BMC Genomics. 2017 May 5;18:351. doi: 10.1186/s12864-017-3722-6 (PMC5420106; doi:10.1186/s12864-017-3722-6)
Supplement: Supplementary file 4 — Exon-intron structure analysis of CpIAA genes. (DOCX 35 kb) [file 12864_2017_3722_MOESM4_ESM.docx]

**Additional file 4**: Exon-intron structure analysis of *CpIAA* genes.


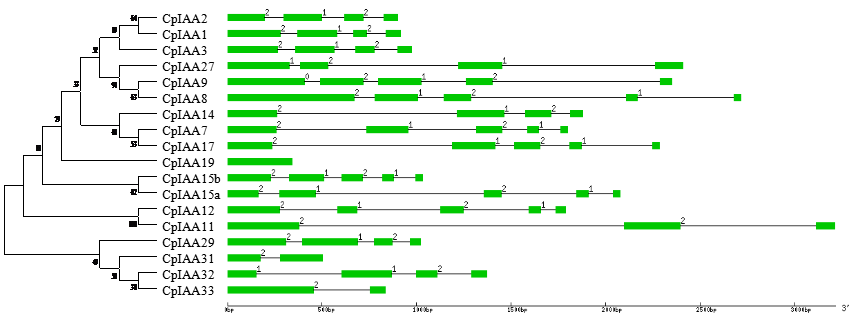


The introns are indicated by gray lines, and the exons are indicated by yellow boxes.
