# Supplementary material for: Genome-wide analysis and characterization of Aux/IAA family genes related to fruit ripening in papaya (Carica papaya L.)
Source: BMC Genomics. 2017 May 5;18:351. doi: 10.1186/s12864-017-3722-6 (PMC5420106; doi:10.1186/s12864-017-3722-6)
Supplement: Supplementary file 5 — The numbers of stress-related cis-elements in the upstream 1.5-kb regions of the CpIAA and AtIAA family genes. (DOCX 13 kb) [file 12864_2017_3722_MOESM5_ESM.docx]

**Additional file 5:** The numbers of stress-related *cis*-elements in the upstream 1.5-kb regions of the *CpIAA* and A*tIAA* family genes.

| gene id | ABRE (YACGTGK) | AuxRE(TGTCTC) | GARE(TCTGTTG) | SARE(TGACG) |
| --- | --- | --- | --- | --- |
| CpIAA2 | 0 | 1 | 2 | 2 |
| CpIAA1 | 1 | 3 | 0 | 1 |
| CpIAA3 | 1 | 3 | 1 | 0 |
| CpIAA27 | 0 | 1 | 1 | 0 |
| CpIAA9 | 0 | 1 | 0 | 1 |
| CpIAA8 | 0 | 2 | 0 | 1 |
| CpIAA14 | 1 | 1 | 1 | 1 |
| CpIAA7 | 1 | 0 | 1 | 0 |
| CpIAA17 | 1 | 1 | 0 | 0 |
| CpIAA19 | NA | NA | NA | NA |
| CpIAA15b | 1 | 0 | 1 | 0 |
| CpIAA15a | 1 | 0 | 0 | 0 |
| CpIAA12 | 0 | 0 | 0 | 3 |
| CpIAA11 | 1 | 2 | 0 | 0 |
| CpIAA29 | 1 | 3 | 0 | 0 |
| CpIAA31 | NA | NA | NA | NA |
| CpIAA32 | NA | NA | NA | NA |
| CpIAA33 | NA | NA | NA | NA |
| AtIAA1 | 0 | 0 | 0 | 0 |
| AtIAA2 | 1 | 0 | 0 | 0 |
| AtIAA3 | 1 | 0 | 0 | 1 |
| AtIAA4 | 0 | 0 | 0 | 0 |
| AtIAA5 | 2 | 1 | 0 | 2 |
| AtIAA6 | 1 | 1 | 1 | 0 |
| AtIAA7 | 1 | 0 | 0 | 0 |
| AtIAA8 | 1 | 2 | 0 | 1 |
| AtIAA9 | 0 | 0 | 0 | 1 |
| AtIAA10 | 0 | 2 | 0 | 0 |
| AtIAA11 | 1 | 0 | 0 | 0 |
| AtIAA12 | 0 | 0 | 0 | 1 |
| AtIAA13 | 0 | 0 | 0 | 0 |
| AtIAA14 | 1 | 0 | 0 | 0 |
| AtIAA15 | 0 | 1 | 0 | 1 |
| AtIAA16 | 0 | 0 | 0 | 1 |
| AtIAA17 | 2 | 0 | 0 | 0 |
| AtIAA18 | 0 | 0 | 1 | 0 |
| AtIAA19 | 2 | 3 | 0 | 0 |
| AtIAA20 | 3 | 1 | 0 | 1 |
| AtIAA26 | 1 | 0 | 0 | 1 |
| AtIAA27 | 0 | 0 | 0 | 0 |
| AtIAA28 | 0 | 2 | 0 | 0 |
| AtIAA29 | 0 | 2 | 0 | 1 |
| AtIAA30 | 1 | 2 | 0 | 2 |
| AtIAA31 | 0 | 0 | 0 | 0 |
| AtIAA32 | 0 | 0 | 0 | 0 |
| AtIAA33 | 0 | 0 | 0 | 2 |
| AtIAA34 | 0 | 1 | 0 | 0 |
