# Supplementary figures and images for: Genome-wide analysis and characterization of Aux/IAA family genes related to fruit ripening in papaya (Carica papaya L.)
Source: BMC Genomics. 2017 May 5;18:351. doi: 10.1186/s12864-017-3722-6 (PMC5420106; doi:10.1186/s12864-017-3722-6)

**Additional file 6:** The expression levels of *AtIAA* genes response to various hormones.


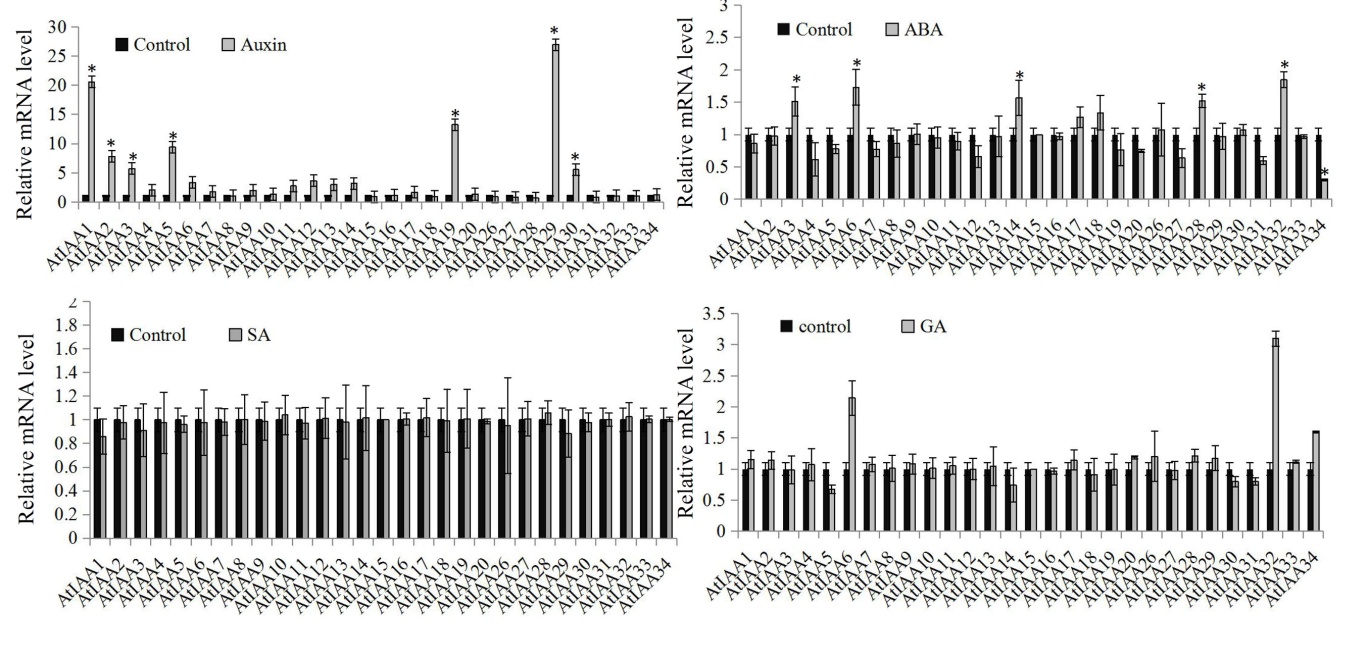

Supplement: Supplementary file 6 — The expression levels of AtIAA genes response to various hormones. (DOCX 294 kb) [file 12864_2017_3722_MOESM6_ESM.docx]
