# Supplementary material for: Genome-wide analysis and characterization of Aux/IAA family genes related to fruit ripening in papaya (Carica papaya L.)
Source: BMC Genomics. 2017 May 5;18:351. doi: 10.1186/s12864-017-3722-6 (PMC5420106; doi:10.1186/s12864-017-3722-6)

**Additional file 7**: a phylogenetic tree to show the relationships of *IAA* genes between papaya and rice.


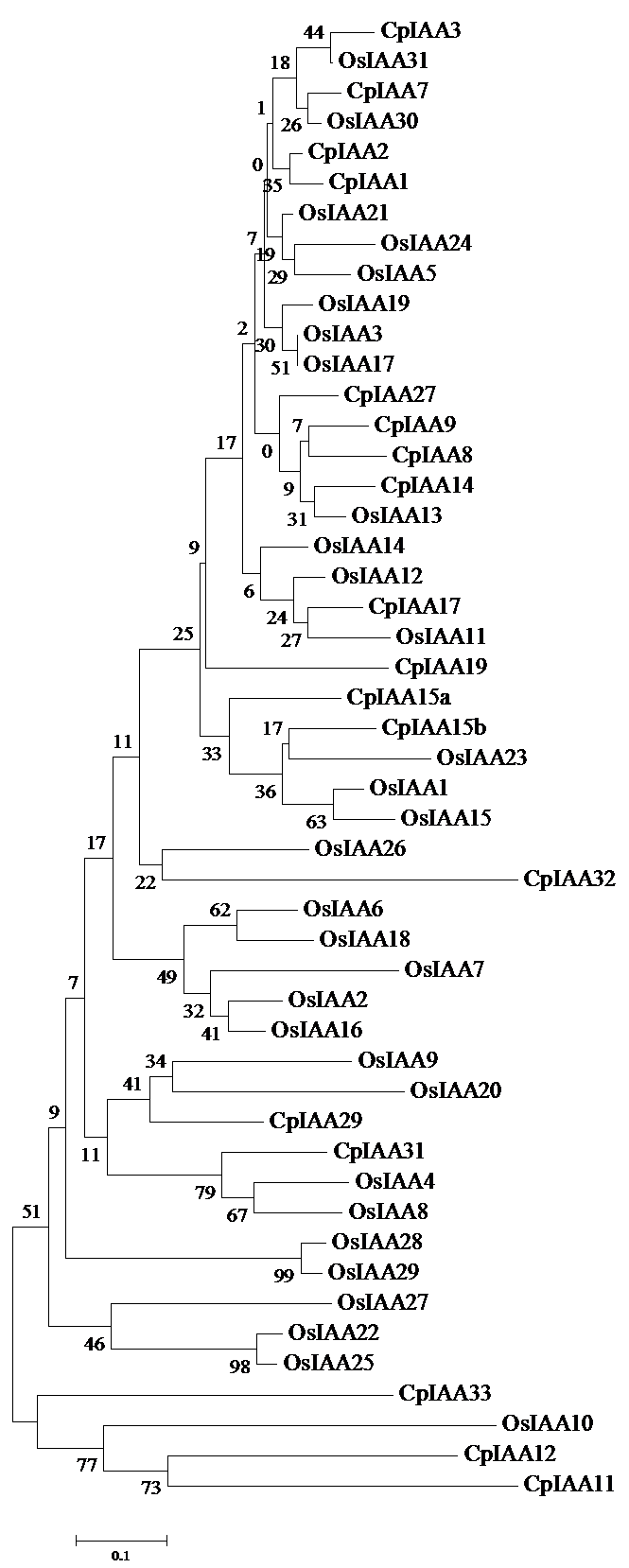

Supplement: Supplementary file 7 — The relationships of IAA genes between papaya and rice. (DOCX 77 kb) [file 12864_2017_3722_MOESM7_ESM.docx]
